# Transcriptome Changes of Skeletal Muscle RNA-Seq Speculates the Mechanism of Postprandial Hyperglycemia in Diabetic Goto-Kakizaki Rats During the Early Stage of T2D

**DOI:** 10.3390/genes10060406

**Published:** 2019-05-28

**Authors:** Wenlu Zhang, Yuhuan Meng, Shuying Fu, Xingsong Li, Zixi Chen, Lizhen Huang, Hongli Du

**Affiliations:** School of Biology and Biological Engineering, South China University of Technology, Guangzhou 510000, China; zhang.wenlu@mail.scut.edu.cn (W.Z.); mengyh@scut.edu.cn (Y.M.); 201610104915@mail.scut.edu.cn (S.F.); 201621034294@mail.scut.edu.cn (X.L.); bizic_chen@mail.scut.edu.cn (Z.C.); Huanglzh@scut.edu.cn (L.H.)

**Keywords:** skeletal muscle, type 2 diabetes (T2D), Goto-Kakizaki (GK) rats, postprandial hyperglycemia, RNA-Seq

## Abstract

To address how skeletal muscle contributes to postprandial hyperglycemia, we performed skeletal muscle transcriptome analysis of diabetic Goto-Kakizaki (GK) and control Wistar rats by RNA sequencing (RNA-Seq). We obtained 600 and 1785 differentially expressed genes in GK rats compared to those Wistar rats at three and four weeks of age, respectively. Specifically, *Tbc1d4*, involved in glucose uptake, was significantly downregulated in the skeletal muscle of GK aged both three and four weeks compared to those of age-matched Wistar rats. *Pdk4*, related to glucose uptake and oxidation, was significantly upregulated in the skeletal muscle of GK aged both three and four weeks compared to that of age-matched Wistar rats. Genes (*Acadl*, *Acsl1* and *Fabp4*) implicated in fatty acid oxidation were significantly upregulated in the skeletal muscle of GK aged four weeks compared to those of age-matched Wistar rats. The overexpression or knockout of *Tbc1d4*, *Pdk4*, *Acadl*, *Acsl1* and *Fabp4* has been reported to change glucose uptake and fatty acid oxidation directly in rodents. By taking the results of previous studies into consideration, we speculated that dysregulation of key dysregulated genes (*Tbc1d4*, *Pdk4*, *Acadl*, *Acsl1* and *Fabp4*) may lead to a decrease in glucose uptake and oxidation, and an increase in fatty acid oxidation in GK skeletal muscle at three and four weeks, which may, in turn, contribute to postprandial hyperglycemia. Our research revealed transcriptome changes in GK skeletal muscle at three and four weeks. *Tbc1d4*, *Acadl*, *Acsl1* and *Fabp4* were found to be associated with early diabetes in GK rats for the first time, which may provide a new scope for pathogenesis of postprandial hyperglycemia.

## 1. Introduction

Type 2 diabetes (T2D), the most prevalent form of diabetes, has affected more than 380 million people worldwide in 2017. Over a quarter of patients suffering from T2D are Chinese (109 million) [1]. Isolated postprandial hyperglycemia is one of the characteristics of diabetes, with the diagnosis criteria requiring a 2-hour plasma glucose concentration ≥11.1 mmol/L and a fasting plasma glucose concentration <7.0 mmol/L, according to a World Health Organization Consultation [2]. However, due to its inconvenient detection method, diagnoses of T2D using isolated postprandial hyperglycemia was not recommended for routine clinical use, with fasting plasma glucose used more commonly in practical diagnoses. Isolated postprandial hyperglycemia is common in Asian diabetes subjects [3], and 46.6% of undiagnosed diabetes subjects in China have isolated postprandial hyperglycemia [4]. The data from a Diabetes Epidemiology Collaborative Analysis of Diagnosis Criteria in Asia (DECODA) study showed that more than 50% of Asian diabetic patients had isolated postprandial hyperglycemia [5]. These findings indicate that isolated postprandial hyperglycemia is one of the main characteristics of Asian or Chinese T2D at the early stage. Therefore, researching the postprandial hyperglycemia mechanism is important for prevention and treatment of T2D. However, there are few researches concerning the molecular mechanisms of postprandial hyperglycemia in T2D patients at the early stage.

Goto-Kakizaki (GK) rats are a non-obese animal model for T2D, generated by selective breeding from Wistar rats with impaired glucose tolerance (IGT) [6,7]. GK rats display postprandial hyperglycemia and glucose intolerance as early as the first week after birth, and show fasting hyperglycemia after four weeks [8], suggesting that postprandial hyperglycemia appears earlier than fasting hyperglycemia in GK rats at early stages. Asian or Chinese T2D patients also have the characteristic of isolated postprandial hyperglycemia at the early stage. Insulin resistance has not been observed in GK rats from one to four weeks of age, while increased insulin sensitivity in GK rats from ages one to three weeks has been reported [8,9]. Thus, postprandial hyperglycemia in GK rats during the early stage of three and four weeks may not result from peripheral insulin resistance. As skeletal muscle is responsible for 70–80% postprandial glucose disposal [10,11], it may play a key role in postprandial hyperglycemia. However, the molecular mechanisms of how skeletal muscle contributes to postprandial hyperglycemia remain unclear. Taken together, GK rats are a suitable model for investigating the molecular mechanisms of skeletal muscle in postprandial hyperglycemia.

To investigate the role of skeletal muscle on postprandial hyperglycemia, diabetic GK and control Wistar rats aged three and four weeks were used in our study. We performed transcriptome and quantitative RT-qPCR analysis of the skeletal muscle of GK and Wistar rats to reveal the molecular mechanisms of postprandial hyperglycemia of T2D. Our study may provide a new scope for dysfunction research of postprandial hyperglycemia in the future.

## 2. Materials and Methods

### 2.1. Animal Breeding and Tissues Collection

In the present study, 10 male rats in each of four groups (GK rats aged three weeks, Wistar rats aged three weeks, GK rats aged four weeks and Wistar rats aged four weeks) were used, totaling 40 subjects. Rats were raised under a 12-hour light-12-hour dark cycle, at a temperature of 20 to 25 °C and 60 ± 5% humidity at the SLAC Laboratory Animal Co., Ltd. (Shanghai, China). All protocols were carried out in accordance with the approved guidelines of the Institutional Animal Care and Use Committee (IACUC) with Ethics certificate IACUC2014029. The rats were not fasted overnight. Blood was collected from the orbital plexus behind the eyeball using EDTA (4 mM final concentration) as an anticoagulant [12,13]. Plasma was obtained from the blood samples by centrifugation (2000×g, 4 °C, 15 min), divided into aliquots, and stored at −80 °C. All rats were killed by cervical dislocation within 2 hours. We harvested the red part of the gastrocnemius muscle from all 10 rats in each group, placed the muscle portion into liquid nitrogen immediately, and then put the portions into a −80 °C freezer for future studies. The rats were weaned at the end of the second week. Six samples from each group were used for RNA sequencing (RNA-Seq).

### 2.2. Measurement of Plasma Glucose and Insulin Concentration 

Random plasma glucose concentrations were measured using an automatic Dry Biochemical Analyzer FUJIFILM DRI-CHEM 7000i (Saitama, Japan). Plasma insulin concentrations were measured using a Thermo scientific Rat Insulin ELISA Kit (Thermo Scientific, Waltham, MA, USA), in accordance with the manufacturer’s instructions.

### 2.3. RNA Extraction, Library Construction and Sequencing

The total RNA of the red part of the gastrocnemius muscle was extracted with TRIzol reagent (Cat#15596-018, Life Technologies, Carlsbad, CA, USA), following the manufacturer’s instructions. The kaiaoK5500^®^ Spectrophotometer (Kaiao, Beijing, China) was used to measure RNA purity. The RNA integrity and concentration were measured using the RNA Nano 6000 Assay Kit (Agilent Technologies, Santa Clara, CA, USA) on the Bioanalyzer 2100 system (Agilent Technologies) (the RNA mass is shown in Supplementary Table S1). Ribosomal RNA was removed using Epicentre Ribo-Zero™ Gold Kits (Epicentre, Madison, WI, USA). Sequencing libraries were subsequently generated following the manufacturer’s recommendations with a varied index label by NEBNext^®^ Ultra™ Directional RNA Library Prep Kit for Illumina (NEB, Beverly, MA, USA). After the preparation of the library, the insert size was assessed by the Agilent Bioanalyzer 2100 system (Agilent Technologies), and qualified using the Taqman fluorescence probe of the AB Step One Plus Real-Time PCR system. Then, RNA-Seq was performed on the Illumina HiSeq X Ten system (Illumina, San Diego, CA, USA) following the HiSeq X Ten User Guide and 150 bp paired-end reads were generated.

### 2.4. Differentially Expressed Genes

After filtering and quality control steps, we used STAR [14] version 020201 to align the clean reads to the reference genome (Rattus norvegicus: Rnor 6.0 version 86, Ensembl). Subsequently, we used Stringtie [15] version 1.3.0 to assemble the transcripts. The fragments per kilobase of exon per million fragments mapped  (FPKM) of all annotated genes were obtained by Ballgown [16] version 2.10.0. Finally, a Bayes-regularized *t*-test, with a false discovery rate (FDR) correction using Cyber-T bayesreg.R [17], was used to obtained the differentially expressed genes (DEGs). FDR < 0.05 was regarded as statistically significant.

### 2.5. Enrichment Analysis of Kyoto Encyclopedia of Genes and Genomes (KEGG) Pathways

The Database for Annotation, Visualization, and Integrated Discovery (DAVID) was used to obtain the Kyoto Encyclopedia of Genes and Genomes (KEGG). A Benjamini correction of *P* value < 0.05 was set as the threshold of significance.

### 2.6. Prediction of the Transcriptional Factors of Key Dysregulated Genes

To investigate the potential upstream regulators that affect gene expression in muscle of GK rats, we used the Match tool in TRANSFANC [18] and JASPAR [19] to predict the transcriptional factors (TFs) of key dysregulated genes. The searching length was 2 kb upstream of each gene locus. The TFs with Relative score < 0.9 predicted by JASPAR were removed. The TFs with core matches <0.9 and matrix matches <0.9 predicted by TRANSFAC were filtered. After filtering, the overlap of TFs in TRANSFAC and JASPAR were regarded as predicted TFs.

### 2.7. Validation of RNA-Seq Results with RT-qPCR 

RT-qPCR analyses were conducted in the LightCycler 96 system (Roche, Basel, Switzerland) to verify the DEGs by SYBR^®^ Premix Ex Taq™ II reagent (TaKaRa, Dalian, China). The methods of 2^−ΔΔCt^ was used to quantify the relative mRNA levels, in which Wistar rats were used as the controls and β-actin (*Actb*) [20,21] was used as an internal reference.

### 2.8. Statistical Analysis

All data were expressed as mean ± SEM unless otherwise stated. Significant differences were assessed using a two-tailed student *t*-test, with *P* values less than 0.05 considered statistically significant.

## 3. Results

### 3.1. The Characterization of Rats

**Figure 1:**
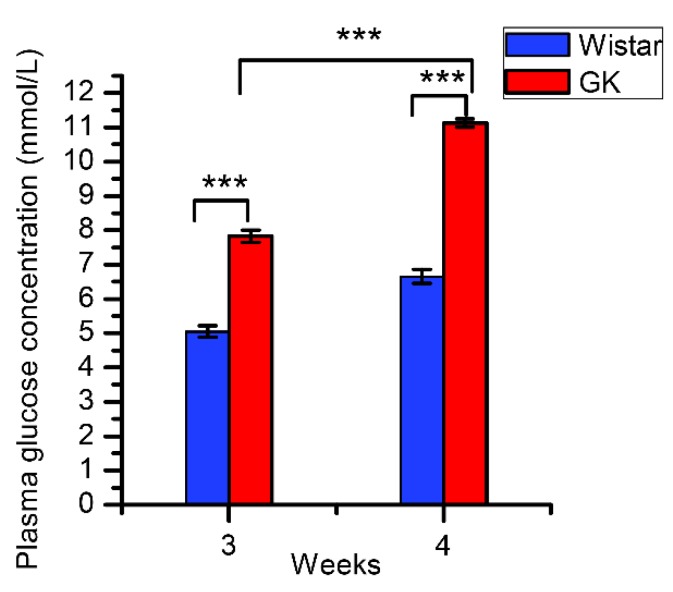
The random plasma glucose concentration of Goto-Kakizaki (GK) and Wistar rats (data are shown as mean ± SEM. *n* = 10, *** *P* < 0.001).

GK random plasma glucose concentration was significantly increased compared to the control at both three and four weeks (Figure 1, *P* < 0.001). Moreover, GK plasma glucose concentration at four weeks was significantly higher than that of GK at three weeks (Figure 1, *P* < 0.001). There was no significant difference in plasma insulin concentration between GK and Wistar rats at both three and four weeks (Supplementary Table S2). The weight of GK rats was similar with Wistar rats at three and four weeks (Supplementary Table S2).

### 3.2. The Differentially Expressed Genes and Enriched Pathways

**Figure 2:**
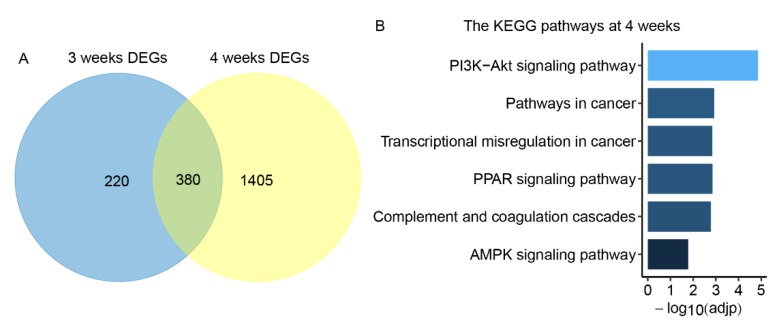
The differentially expressed genes (DEGs) and enriched pathways in GK skeletal muscle. (**A**) Venn diagram of differentially expressed genes in GK skeletal muscle compared to age-matched Wistar rats at three and four weeks of age. (**B**) The Kyoto Encyclopedia of Genes and Genomes (KEGG) pathways enriched in GK skeletal muscle at four weeks (Benjamini correction of *P* < 0.05). Adjp represents the Benjamini correction of *P* values.

In total, 12,300 genes were detected with expression (mean FPKM > 1) in GK and Wistar skeletal muscle at three and four weeks. This detected gene number was similar to that of a previous study [22]. Among the detected genes, and in comparison to the Wistar rats, 600 and 1785 differentially expressed genes (DEGs) were obtained from GK skeletal muscle at three and four weeks, respectively (FDR < 0.05, Supplementary Table S3). Of these DEGs, there were 380 overlap DEGs at both three and four weeks. We found 220 DEGs were specific at three weeks and 1405 DEGs were specific at four weeks (Figure 2A). Additionally, 273 and 1027 DEGs were upregulated at three and four weeks, respectively, while 327 and 758 DEGs were downregulated at three and four weeks, respectively (Supplementary Table S3). A KEGG signaling pathway enrichment analysis with DEGs was conducted using DAVID. There was no enriched pathway at three weeks (Benjamini correction of *P* < 0.05). In contrast, six KEGG pathways were enriched at four weeks (Figure 2B, Benjamini correction of *P* < 0.05) and the PI3K-Akt signaling pathway was ranked first with the lowest Benjamini corrected *P* value. These results indicate the dynamic diabetic processes of GK rats from three to four weeks. It has been reported that mice with skeletal muscle which specifically overexpresses *Nr4a1* (orphan nuclear receptor 4A1, also known as Nur77) have enhanced fatty acid oxidation [23]. This suggests that upregulation of the gene *Nr4a1* in GK skeletal muscle at four weeks—which was enriched in the PI3K-Akt signaling pathway—may facilitate fatty acid oxidation in GK skeletal muscle compared to that of age-matched Wistar rats. 

### 3.3. The Differentially Expressed Genes Related to Glucose Uptake and Oxidation

Since glucose transport is the rate-limiting step for glucose uptake and utilization in muscle [24,25,26], and glucose transport, uptake and oxidation are potentially implicated in the regulation of glucose homeostasis, we focused on the genes related to glucose transport, uptake and oxidation. 

**Figure 3:**
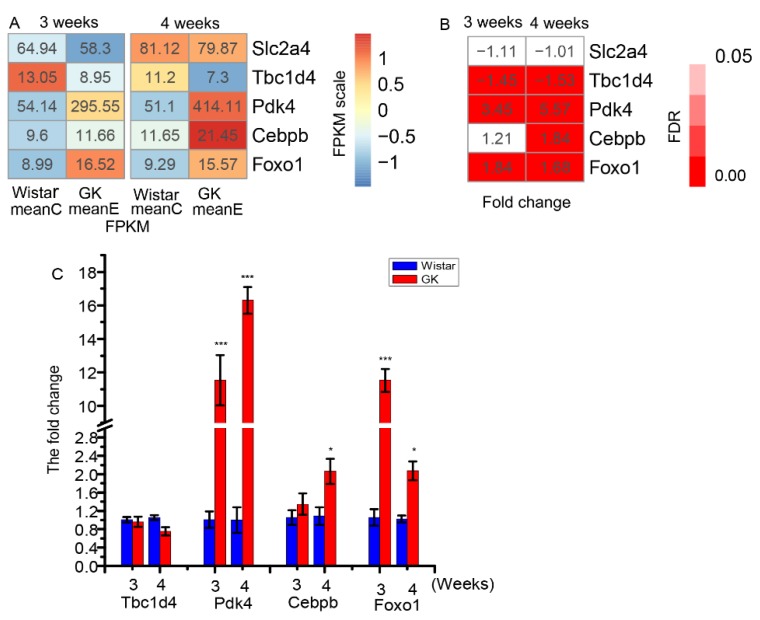
The differentially expressed genes related to glucose uptake and oxidation. (**A**) The mean fragments per kilobase of exon per million fragments mapped  (FPKM) of *Pdk4*, *Slc2a4*, *Tbc1d4*, *Cebpb* and *Foxo1* in skeletal muscle of GK and Wistar rats. (**B**) The fold change (meanE/meanC) and corresponding false discovery rate (FDR) values of *Pdk4*, *Slc2a4*, *Tbc1d4*, *Cebpb* and *Foxo1* in GK rats in comparison with those of Wistar rats. (**C**) The fold change (2^−ΔΔCt^) of GK genes *Pdk4*, *Tbc1d4*, *Cebpb* and *Foxo1* expression compared with Wistar rats by RT-qPCR (data are shown as mean ± SEM. *n* = 6, * *P* < 0.05, *** *P* < 0.001). MeanC represents the mean FPKM of Wistar rats, while meanE represents the mean FPKM of GK rats.

There was no significant difference in the expression of *Slc2a4* (glucose transporter 4, also termed as Glut4) between GK and Wistar skeletal muscle at three or four weeks (Figure 3A,B). The FPKM of gene *Tbc1d4* (TBC1 domain family, member 4, also termed AS160) was significantly reduced in GK skeletal muscle compared to that of age-matched Wistar rats at both three and four weeks (FDR < 0.05 and FDR < 0.001, respectively) (Figure 3A,B). The relative expression of *Tbc1d4* was verified as downregulated without significant differences by RT-qPCR (Figure 3C).

The expression of *Pdk4* (pyruvate dehydrogenase kinase 4) was significantly increased in GK skeletal muscle compared to that of age-matched Wistar rats at three and four weeks (FDR < 0.001) (Figure 3A and 3B), which was verified as upregulated by RT-qPCR (Figure 3C) with significant difference (Figure 3C). The expression of two confirmed *Pdk4* transcription factors, *Cebpb* (CCAAT/enhancer-binding protein β, C/EBPβ) and *Foxo1* (forkhead box O1), were increased in GK skeletal muscle in comparison to age-matched Wistar rats at three and four weeks (Figure 3), indicating that the two *Pdk4* transcription factors, *Cebpb* and *Foxo1*, may be responsible for *Pdk4* upregulation.

As glycolysis is an important pathway for glucose oxidation, we also focused on the genes related to glycolysis. The expression of genes related to glycolysis, including *Hk2* (hexokinase 2), *Pfkm* (phosphofructokinase, muscle), and *Pkm* (pyruvate kinase) showed no significant difference between GK and Wistar rats (Supplementary Table S4).

### 3.4. The Differentially Expressed Genes Related to Fatty Acid Oxidation

**Figure 4:**
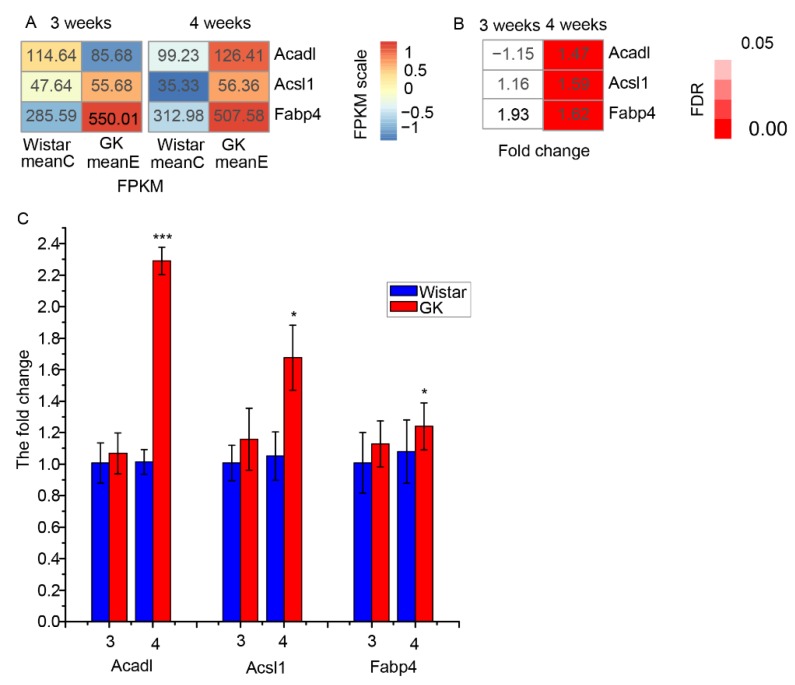
The differentially expressed genes related to fatty acid oxidation. (**A**) The mean FPKM of *Acadl*, *Acsl1* and *Fabp4* in skeletal muscle of GK and Wistar rats. (**B**) The fold change (meanE /meanC) and corresponding FDR values of *Acadl*, *Acsl1* and *Fabp4* in GK rats in comparison with those of Wistar rats. (**C**) The fold change (2^−ΔΔCt^) of GK genes *Acadl*, *Acsl1* and *Fabp4* expression compared with Wistar rats by RT-qPCR (data are shown as mean ± SEM. *n* = 6, * *P* < 0.05, *** *P* < 0.001). MeanC represents the mean FPKM of Wistar rats, while meanE represents the mean FPKM of GK rats.

As Randle et al. pointed out, increased oxidation of fatty acids could repress glucose oxidation [27,28]. Genes (*Acadl*, *Acsl1*, *Fabp4* and *Cpt1a*) related to fatty acid oxidation may be implicated in the regulation of glucose oxidation. Among those genes, the expression of *Acadl* (acyl-CoA dehydrogenase, long chain), *Acsl1* (acyl-CoA synthetase long-chain family member 1) and *Fabp4* (fatty acid binding protein 4) showed no significant difference in GK skeletal muscle at three weeks compared to that of age-matched Wistar rats (Figure 4A,B), and were significantly increased at four weeks, as verified by RT-qPCR (Figure 4C). Cpt1a (carnitine palmitoyltransferase 1A), encoded by *Cpt1a* and responsible for transportation of long-chain fatty acids into mitochondria, was significantly increased in GK skeletal muscle in comparison to age-matched Wistar rats at three and four weeks (mean FPKM < 6, Supplementary Table S4). Other genes involved in the oxidation of fatty acids did not show significant differences between the GK and Wistar rat groups (Supplementary Table S4).

### 3.5. The Predicted Transcriptional Factors of Key Dysregulated Genes

**Figure 5:**
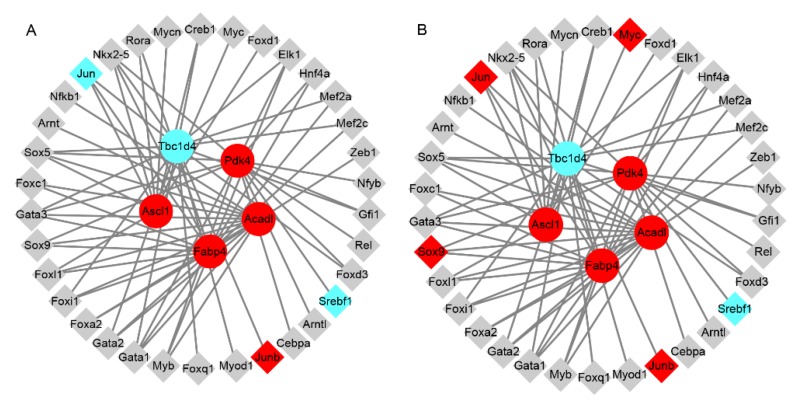
The network of *Acadl*, *Acsl1*, *Fabp4*, *Pdk4* and *Tbc1d4* and their dysregulated transcription factors (TFs). (**A**) The network of *Acadl*, *Acsl1*, *Fabp4*, *Pdk4* and *Tbc1d4* and their dysregulated TFs at three weeks. (**B**) The network of *Acadl*, *Acsl1*, *Fabp4*, *Pdk4* and *Tbc1d4* and their dysregulated TFs at four weeks. Grey represents the genes expressed without significant difference; cyan represents the downregulated expression of genes; and red represents the upregulated expression of genes. The circled genes represent the target genes of the TFs, while the diamonds represent the individual TFs.

To investigate the factors affecting the expression of key dysregulated genes, we predicted the transcriptional factor (TFs) of genes *Acadl*, *Acsl1*, *Fabp4*, *Pdk4* and *Tbcld4*. The TFs predicted by TRANSFAC and JASPAR are shown in Supplementary Table S7. There were 12, 9, 17, 16 and 16 overlapping TFs of *Acadl*, *Acsl1*, *Fabp4*, *Pdk4* and *Tbcld4*, respectively, according to TRANSFAC and JASPAR (Supplementary Table S7). The overlap TFs with dysregulated expression at three weeks and four weeks were Jun (proto-oncogene, AP-1 transcription factor subunit), Myc (proto-oncogene, bHLH transcription factor), Sox9 (SRY box 9), Srebf1 (sterol regulatory element binding transcription factor 1) and Junb (JunB proto-oncogene, AP-1 transcription factor subunit). Jun, which was the predicted TF of *Acsl1*, *Fabp4* and *Tbc1d4*, was significantly downregulated in GK skeletal muscle compared to that of age-matched Wistar rats at three weeks, but was significantly upregulated in GK skeletal muscle at four weeks compared to age-matched Wistar rats (Figure 5). Myc was the predicted TF of *Acsl1* and was significantly upregulated in GK skeletal muscle compared to that of age-matched Wistar rats at four weeks, but with no significant difference at three weeks. Sox9, which was the predicted TF of *Fabp4* and *Pdk4*, was significantly upregulated in GK skeletal muscle compared to Wistar rats at four weeks, but was without significant difference at three weeks. Srebf1 was significantly downregulated in GK skeletal muscle compared to that of age-matched Wistar rats at three and four weeks, and was the predicted TF of *Pdk4*. Junb was the predicted TF of *Tbc1d4* and was significantly upregulated in GK skeletal muscle compared to that of age-matched Wistar rats at three and four weeks. Therefore, dysregulation of the predicted TFs may lead to the dysregulated expression of *Acadl*, *Acsl1*, *Fabp4*, *Pdk4* and *Tbcld4*, although further study is needed to verify this.

## 4. Discussion

It is known that postprandial hyperglycemia is important in whole-body glycemic control in T2D patients [29,30]. In addition, postprandial hyperglycemia is one of the early events in T2D patients [31]. As such, it is essential to reduce postprandial hyperglycemia of T2D patients [32]. Since GK rats are a suitable model for investigating the mechanism of postprandial hyperglycemia in skeletal muscle, we performed transcriptome analysis on the skeletal muscle of GK and Wistar (control) rats by RNA-Seq. From this analysis, 600 and 1785 DEGs were obtained from the skeletal muscle of GK rats at three and four weeks, respectively, compared to the Wistar rats. In considering previous studies, we predicted that dysregulated expression of key dysregulated genes (*Acadl*, *Acsl1*, *Fabp4*, *Pdk4* and *Tbcld4*) could lead to decreased glucose uptake and oxidation, as well as enhanced fatty acid oxidation, in GK skeletal muscle at three and four weeks. These dysregulated metabolic processes may lead to the accumulation of glucose in the blood of GK individuals, which in turn may result in postprandial hyperglycemia in GK rats during an early stage (three and four weeks).

Glucose uptake is a complex process that involves a number of genes. Among these genes, *Slc2a4* encodes the transporter protein Glut4. Glut4 mediates postprandial glucose transport in skeletal muscle [33,34], and is also necessary for glucose homeostasis in muscles [35,36,37,38]. In fact, muscle-specific *Slc2a4* knockout mice have been shown to exhibit significantly reduced glucose uptake in their muscles [39,40,41]. It has also been reported that Tbc1d4, encoded by the gene *Tbc1d4*, is responsible for regulating glucose transport [42,43]. Whole-body *Tbc1d4* knockout mice have exhibited significantly decreased insulin-stimulated glucose uptake in their skeletal muscle [44,45]. The overexpression of *Pdk4* could decrease glucose uptake, and knockdown of *Pdk4* has been shown to increase glucose uptake in neonatal myocytes [46]. Glycogen synthesis is also implicated in glucose disposal in skeletal muscle in the postprandial state [47], which may influence the homeostasis of glucose. *Gsk3b* (glycogen synthase kinase 3 beta) and *Gys1* (glycogen synthase 1) play key roles in glycogen synthesis [48,49]. It has been reported that insulin-stimulated glucose uptake is markedly decreased in the skeletal muscle of GK rats weighing 280–300 g [50,51,52]. In addition, a study on *Pdk4* expression has shown that it is significantly higher in GK skeletal muscle from 4–12 weeks [53], which is consistent with our results. In the present study, there was no expression difference in *Slc2a4* (Figure 3), *Gsk3b* or *Gys1* (Supplementary Table S4) in GK and Wistar skeletal muscle at three and four weeks. Therefore, the significantly decreased *Tbc1d4* expression and the significantly increased *Pdk4* expression in GK skeletal muscle—compared to that of age-matched Wistar rats—may have led to the observed decrease in glucose uptake of GK skeletal muscle at three and four weeks. Further study is needed to confirm the reduced glucose uptake of GK skeletal muscle at three and four weeks.

It is well known that there is metabolic competition between fatty acid oxidation and glucose oxidation, wherein increased fatty acid oxidation can inhibit glucose oxidation in heart and skeletal muscle [27,28]. The genes (*Acadl*, *Acsl1* and *Fabp4*) related to fatty acid oxidation are also involved in regulating glucose oxidation, which would therefore affect the glucose content of blood [12,54]. Herein, the expression of genes (*Acadl*, *Acsl1*, *Fabp4* and *Cpt1a*) related to fatty acid oxidation have exhibited an increase in GK skeletal muscle compared to that of age-matched Wistar rats at three weeks, and even more notably at four weeks. It has been reported that muscle-specific *Acadl* knockout mice exhibit reduced fatty acid oxidation [55]. Furthermore, skeletal muscle-specific deficiency *Acsl1* mice have been shown to exhibit a significant decrease in fatty acid oxidation and lower plasma glucose concentration [54]. The gene product of *Fabp4* has been shown to band and transport intracellular long-chain fatty acids. Whole-body *Fabp4* knockout mice exhibit a reduction in free fatty acid release and a reduced plasma glucose level [12,13]. It has been suggested that overexpression of *Cpt1a* leads to enhanced fatty acid oxidation in hepatocytes, β-cells and muscle cells [56,57,58,59]. Besides regulating glucose uptake, *Pdk4* is also implicated in regulating oxidation of glucose and fatty acids, with upregulated expression of *Pdk4* reducing glucose oxidation and enhancing fatty acid oxidation in myocardium and skeletal muscle [60]. It has been demonstrated that skeletal muscle-specific overexpression of *Nr4a1* facilitates fatty acid oxidation in the skeletal muscle of mice [23]. Reduced glucose oxidation in the skeletal muscle of GK rats aged 8–10 weeks has also been reported [51,52]. Moreover, it has been reported that the muscle lipid contents in GK rats is lower than that of Wistar rats aged four weeks and seven months [9,61]. Thus, the increased expression of key dysregulated genes (*Acadl*, *Acsl1*, *Fabp4* and *Pdk4*) in GK skeletal muscle—in comparison to the age-matched Wistar rats—may have resulted in the enhanced fatty acid oxidation and reduced glucose oxidation in the GK skeletal muscle at three and four weeks. Additionally, the increased *Nr4a1* expression in GK skeletal muscle compared to that of Wistar rats may be associated with the enhanced fatty acid oxidation and reduced glucose oxidation in GK skeletal muscle at four weeks. Further study is needed to confirm the increase in fatty acid oxidation and reduction in glucose oxidation in the skeletal muscle of GK rats aged three and four weeks.

**Figure 6:**
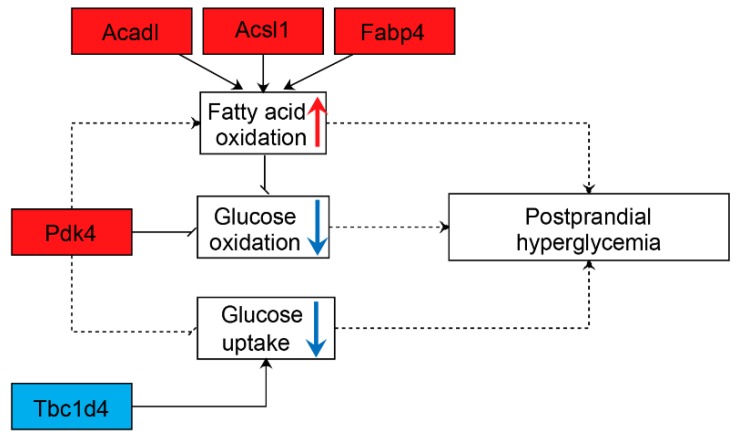
The putative mechanism of postprandial hyperglycemia in GK skeletal muscle during the early stage of three and four weeks. The downregulated expression of *Tbc1d4* would reduce the uptake of glucose. The upregulated expression of *Acadl*, *Acsl1* and *Fabp4* could contribute to enhanced fatty acid oxidation, which could repress the oxidation of glucose. Moreover, increased *Pdk4* expression may not only reduce glucose uptake, but could also reduce glucose oxidation and enhance fatty acid oxidation. Reduced glucose uptake and oxidation, as well as enhanced fatty acid oxidation, may result in postprandial hyperglycemia. The red boxes represent upregulated expression, while the blue box represents downregulated expression. The upward red arrow represents an increase, while the downward blue arrow represents a reduction. The dotted lines indicate that the precise role is unclear.

By integrating previous studies and the results of our analysis, a putative mechanism of postprandial hyperglycemia in GK skeletal muscle during the early stage of three and four weeks is proposed in Figure 6. The putative mechanism suggests that the dysregulated expression of certain genes (*Tbcld4*, *Pdk4*, *Acadl*, *Acsl1*, and *Fabp4*) may result in increased fatty acid oxidation and reduced glucose uptake and oxidation in GK skeletal muscle during the early stage of three and four weeks. This may result in the accumulation of glucose in the blood of GK individuals and, in turn, postprandial hyperglycemia in those rats.

## 5. Conclusions

In the present study, we revealed transcriptome changes in GK skeletal muscle during the early stages of three and four weeks and found that the expression of *Tbc1d4*, *Pdk4*, *Acadl*, *Acsl1* and *Fabp4* were dysregulated. This is the first study that suggests *Tbc1d4*, *Acadl*, *Acsl1* and *Fabp4* are associated with early diabetes in GK rats, which may provide a new scope for pathogenesis of postprandial hyperglycemia and controlling postprandial glucose levels in T2D patients at early stages.

## Data Availability

The RNA-Seq data used to support this study is uploaded to NCBI, the SRA accession is PRJNA540098.

## Supplementary Materials

The following are available online at https://www.mdpi.com/2073-4425/10/6/406/s1, Table S1 The information of RNA mass, Table S2 The insulin concentration and body weight of rats. (Data are shown as mean ± SEM. *n* = 10), Table S3 The differentially expressed genes in GK rats compared with Wistar rats at three and four weeks, Table S4 The details of genes related to glycolysis, fatty acid oxidation and glycogen synthesis at three and four weeks, Table S5 The TFs predicted by TRANSFAC and JASPAR.
